# Development of Different Methods for Preparing *Acinetobacter baumannii* Outer Membrane Vesicles Vaccine: Impact of Preparation Method on Protective Efficacy

**DOI:** 10.3389/fimmu.2020.01069

**Published:** 2020-06-23

**Authors:** Sun Li, Da-Qun Chen, Lu Ji, Si Sun, Zhe Jin, Zi-Li Jin, Hong-Wu Sun, Hao Zeng, Wei-Jun Zhang, Dong-Shui Lu, Ping Luo, An-Ni Zhao, Jiao Luo, Quan-Ming Zou, Hai-Bo Li

**Affiliations:** ^1^Department of Microbiology and Biochemical Pharmacy, National Engineering Research Center of Immunological Products, College of Pharmacy, Third Military Medical University, Chongqing, China; ^2^Department of Clinical Epidemiology, Leiden University Medical Center, Leiden, Netherlands

**Keywords:** *Acinetobacter baumannii*, preparation method, protective efficacy, vaccine, intranasal administration, intramuscular administration

## Abstract

*Acinetobacter baumannii* (*A. baumannii*) is becoming a common global concern due to the emergence of multi-drug or pan-drug resistant strains. Confronting the issue of antimicrobial resistance by developing vaccines against the resistant pathogen is becoming a common strategy. In this study, different methods for preparing *A. baumannii* outer membrane vesicles (AbOMVs) vaccines were developed. sOMV (spontaneously released AbOMV) was extracted from the culture supernatant, while SuOMV (sucrose-extracted AbOMV) and nOMV (native AbOMV) were prepared from the bacterial cells. Three AbOMVs exhibited significant differences in yield, particle size, protein composition, and LPS/DNA content. To compare the protective efficacy of the three AbOMVs, groups of mice were immunized either intramuscularly or intranasally with each AbOMV. Vaccination via both routes conferred significant protection against lethal and sub-lethal *A. baumannii* challenge. Moreover, intranasal vaccination provided more robust protection, which may be attributed to the induction of significant sIgA response in mucosal sites. Among the three AbOMVs, SuOMV elicited the highest level of protective immunity against *A. baumannii* infection, whether intramuscular or intranasal immunization, which was characterized by the expression of the most profound specific serum IgG or mucosal sIgA. Taken together, the preparation method had a significant effect on the yield, morphology, and composition of AbOMVs, that further influenced the protective effect against *A. baumannii* infection.

## Introduction

*Acinetobacter baumannii* (*A. baumannii*) is a gram-negative, aerobic bacterium, which is generally harmless to healthy individuals, but can cause serious infections such as ventilator-associated pneumonia, bloodstream infection, and wound infections in hospitalized, severely unwell patients on intensive care units (1–3). Due to intrinsic or acquired resistance to a wide range of antibiotics, resulting in multidrug-resistant (MDR) or even pandrug-resistant (PDR) strains (4), *A. baumannii* has emerged as one of the most challenging pathogens to effectively treat (5, 6). The mortality rates associated with *A. baumannii* infection has reported to be 26.0–55.7% (7), and thus effective prevention or treatment procedures are urgently needed.

Vaccines are the most efficient and cost-effective way to control infectious diseases. Different kinds of vaccines have been formulated aiming at protecting against *A. baumannii* infection. Inactivated whole cells (IWC) (8), outer membrane complex (OMC) (9), outer membrane vesicles (OMVs) (10, 11), and several outer membrane proteins including OmpA (12), Omp22 (13), and OmpK (14) have been identified as vaccine candidates through active and passive immunizations of experimental animals.

OMVs are highly immunogenic nano-sized spherical structures enriched with outer membrane proteins, and are considered as vaccine candidates against derived pathogenic bacteria (15, 16). OMV vaccines against serogroup B strains of *Neisseria meningitidis* (MenB) have been licensed in Norway, Cuba, and New Zealand, which led to the substantial reduction of local cases of invasive meningococcal disease (17, 18). Several methods have been developed to prepare OMVs. Gram-negative bacteria secrete OMVs spontaneously to the culture medium in the process of growth, and OMVs could be enriched and isolated from the cell culture supernatant (19). Other methods to obtain OMVs from bacterial cells included using detergents and/or chelating agents to promote the production of the OMVs (20, 21), always with a higher yield than isolation from the culture supernatant (22). In addition to yield, it was proven that the MenB OMVs obtained by varied methods exhibited differences in morphology, protein composition, toxicity, immunogenicity, and even in serum bactericidal activity (SBA) (23–25).

The most studied method to obtain *A. baumannii* OMV (AbOMV) so far has been isolation by the combination of ultrafiltration and ultracentrifugation from the cell culture supernatants (10, 26, 27). Though AbOMV isolated from the above method was immunogenic and protective, the yield of AbOMV is generally low. In the current study, novel methods were established to prepare AbOMV from bacterial cells. The AbOMVs obtained from different methods were analyzed for yield, morphology, surface charge, protein composition, and immunogenicity. The protective efficacy against the infection of *A. baumannii* was also evaluated and the mechanism was explored with the objective to provide a basis for the selection of the best approach for AbOMV vaccine development.

## Materials and Methods

### Ethics Statement

All animal care and protocols were performed in accordance with the Regulations for the Administration of Affairs Concerning Experimental Animals approved by the State Council of People's Republic of China. All animal experiments were approved by the Animal Ethical and Experimental Committee of the Third Military Medical University. Well-trained and skilled animal care personnel participated in the current study to minimize the suffering of animals.

### Animals and Bacteria Strains

SPF female C57BL/6J mice of 6–8 weeks were purchased from the Experimental Animal Center of the Third Military Medical University. All mice were kept under pathogen-free conditions in the animal center of the Third Military Medical University (Chongqing, China). *A. baumannii* ATCC17978 and LAC-4 were kindly provided by Prof. Yun Shi (West China Hospital, Sichuan University).

### Reagents

RPMI 1640(SH30809.01), TRYPSIN 0.25% Solution (SH30042.01), and PBS buffer (SH30256.01B) were purchased from HyClone; Penicillin-streptomycin solution (100 × , C0222), PE anti-mouse CD40(124609), APC anti-mouse CD86 (105011), PerCP/Cy5.5 anti-mouse CD80 (104721), and FITC anti-mouse CD11c(117305) were purchased from Beyotime; Murine M-CSF(315-02-10 μg) and IL-4(214-14-20 μg) were purchased from PEPROTECH; FBS (P30-3302) was purchased from PAN; Goat Anti-Mouse IgA alpha chain (HRP) (ab97235), Goat Anti-Mouse IgG1 heavy chain (HRP) (ab97240), and Goat Anti-Mouse IgG2a heavy chain (HRP) (ab97245) were purchased from Abcam; Mouse TNF-α ELISA kit (1217202), Mouse IL-12p70 Elisa kit (1211202), Mouse IL-10 ELISA kit (1211002), and Mouse IL-6 ELISA kit (1210602) were purchased from Dakewe; Lowry protein concentration assay kit (PC0030), DAB color development kit (DA1010), and KDO (2-keto-3-deoxyoctane, K2755) was purchased from Solarbio; Quant-iT™ PicoGreen® dsDNA Reagent (P7581) was purchased from Thermo (Invitrogen).

### Preparation of AbOMVs

*A. baumannii* sOMV (spontaneously released OMV) were prepared as described previously with slight modification (26). Briefly, the *A. baumannii* 17978 was grown to late log phase in 10 liters LB broth with shaking at 37°C. After centrifugation at 20,000 × g for 30 min, the supernatant was filtered through a 0.22 μm membrane and further concentrated by ultrafiltration with a 100 kD hollow fiber using the QuixStand Benchtop System (GE Healthcare, USA). The retained sample was filtered (0.22 μm) once more and subjected to ultracentrifugation at 12,5000 × g for 2 h at 4°C. The pellets were applied to the bottom of discontinuous density gradient centrifugation using OptiPrep^TM^ (45%, 35%, 30%, 25%, 20%, 15%) at 150,000 × g for 5 h at 4°C. *A. baumannii* sOMV was collected and finally diluted with PBS.

*A. baumannii* nOMV (native OMV) was prepared from bacterial cells cultured in 10 liters of LB broth. The cells were suspended in Buffer (10 mM Tris-HCl, 10 mM EDTA, 150 mM NaCl, pH7.4), 2.5 times wet weight, and incubated at 56°C for 1 h. After being cooled to 4°C, the suspension was sheared at 18000 rpm for 5 min in a High-speed dispersator (XHF-D, Ningbo Scientz, China) in an ice bath (19, 28). The suspension was centrifuged first at 5,000 × g for 20 min, and then at 30,000 × g for 20 min. The resulting supernatant was centrifuged at 150,000 × g for 2 h. Finally, *A. baumannii* nOMV was washed and diluted with PBS.

*A. baumannii* SuOMV (sucrose-extracted OMV) were prepared based on isopycnic sucrose gradient centrifugation followed by rupture of the bacteria (29). Briefly, the *A. baumannii* 17978 was grown to late log phase in 5 liters LB broth with shaking at 37°C. Bacterial cells were harvested by centrifugation at 5,000 × g for 30 min at 4°C. The wet weight of the harvested cells was determined, and the cell pellets were suspended with the precooling Buffer A (0.75 M sucrose, 10 mM Tris-HCl, pH 7.8). The ratio of buffer to bacterial cells was 20:1 (v/w). After dropwise addition of 10 mg/ml of lysozyme in the ice bath, the cell suspension was diluted with 2 times volume of cold 1.5 mM EDTA. The sample was then sonicated on ice (10 × 5-s bursts at 20% maximum power) using a sonicator (Ningbo Scientz, China). Unlysed cells were removed by centrifugation at 1,200 × g for 15 min. After ultracentrifugation (360,000 × g, 2 h, 4°C) the extraction was suspended with Buffer B (0.25 M sucrose, 10 mM Tris-HCl, 1 mM EDTA, pH 7.8) to the same volume. The suspension was then centrifuged (360,000 × g, 2 h, 4°C) as before, and the pellet was carefully suspended in cold Buffer C (25% sucrose, 5 mM EDTA, pH 7.5). *A. baumannii* SuOMV were further purified by sucrose density gradient (55, 50, 45, 40, 35, 30% sucrose in 5 mM EDTA, pH 7.5) centrifugation (250,000 × g for 16 h) and diluted with PBS (30).

### Analysis Procedure

Total protein of each AbOMV was used to estimate OMV yield and the concentration of total protein was measured by the Lowry method. Protein composition was determined by 12% SDS-PAGE stained with Coomassie blue and Linear Trap Quadropole (LTQ) Orbitrap Velos Pro Mass Spectrometry (Thermo Fisher Scientific) (31). Residual lipopolysaccharide (LPS) concentration was determined with the KDO assay (32). DNA quantification assays were performed using Quant-iT™ PicoGreen® dsDNA reagent according to the manufacturer's recommendation.

### Transmission Electron Microscopy (TEM)

The morphology of AbOMVs was investigated by TEM. Each AbOMV was diluted with deionized water to 100 μg/mL, dropped onto the copper mesh, stained with uranyl acetate for 30 s, and vacuum dried for 1 h (33). TEM images were taken by a JEOL JEM-1230 (JEOL Ltd, Tokyo, Japan).

### Generation of BMDCs and FACS Assay

Mouse bone marrow cells were isolated from tibia and femurs, and then were cultured in RPMI 1640 medium containing 10% FCS, recombinant murine GM-CSF (6 ng/mL), and IL-4 (20 ng/mL) at 37°C, 5% CO_2_. The non-adherent cells were removed on day 5 and adherent cells were cultured in a fresh complete medium for another 2 days (34). BMDCs (4 × 10^6^ cells) were stimulated with either AbOMV (200 ng) or PBS for 48 h. After three washes, the cells were incubated with FITC-αCD11c, PE-αCD40, APC-αCD80, and PerCP/Cy5.5-αCD86 in the dark for 30 min at 4°C. Flow cytometry data were acquired with a FACS Canto II (BD Biosciences).

### Cytokines Assay

BMDCs (4 × 10^6^ cells/well in 4 mL RMPI-1640 medium) were stimulated with either AbOMV (0.8 μg/well) or PBS for 48 h. Concentrations of TNF-α, IL-6, IL-1β, IL-10, IFN-γ, and IL-12p70 in the culture supernatants were detected by ELISA kit.

### Animal Immunization

For the different delivery routes, mice were divided randomly into two sections. In the first section, the mice were immunized intramuscularly three times at intervals of 2 weeks with 20 μg AbOMV absorbed to aluminum phosphate adjuvant (500 μg/mouse). In the second section, the mice were anesthetized with pentobarbital sodium (50 mg/kg) and then intranasally immunized with 10 μL PBS containing 20 μg AbOMV. Saliva, vaginal wash, and sera were collected for further analysis.

### Antibody Production Assay

Antibody levels were measured by standard indirect ELISA as previously described (35). Briefly, 96-well flat bottom microtiter plates were each coated with AbOMV and incubated overnight at 4°C. After blocking with 5% BSA in PBST, the 1:1,000 pre-diluted sera samples were added to 96-well microtiter plates followed by a 1:1 gradient dilution and incubated for 40 min at 37°C. HRP-conjugated goat anti-mouse IgG, IgG1, or IgG2a was then used as a secondary antibody to determine antigen-specific IgG or IgG subtype level. Each well had 100 μL TMB substrate solution added and then was kept in darkness for 20 min. The reaction was finally quenched by stop solution (2M H_2_SO_4_). The optical density (OD) was measured at 450 nm in a microplate reader (Bio-Rad). The sIgA level in the saliva and vaginal wash was measured by ELISA as described above.

### Challenges and Survival Rates

Two weeks following the last immunization, mice were anesthetized with an intraperitoneal injection of pentobarbital sodium (50 mg/kg) and then challenged with 2 × 10^7^ (lethal dose) or 5 × 10^6^ (sublethal dose) colony-forming unit (CFU) of LAC-4 in 20 μl PBS by non-invasive intratracheal inoculation as previously described (36). The challenge dose was confirmed by CFU counts of serial 10-fold dilutions on tryptone soy agar (TSA). The survival rate of control and immunized mice were monitored daily for 7 days (*n* = 10). Moreover, bacteria burdens in the mice lung and blood were determined at 24 h post sublethal challenge (*n* = 5) (37).

### Statistical Analysis

All statistics were analyzed by Graph Pad Prism7.0. Continuous variables were expressed as means ± standard deviation (SD). Inter-group differences were analyzed by one-way ANOVA, and differences with a *p* ≤ 0.05 means the result was statistically significant. If inter-group differences were statistically significant, pairwise comparisons were further performed by a Bonferroni test, with the significant level α'= α/k (k is the number of comparison times).

## Results

### Preparation and Characterization of AbOMVs

**Figure 1:**
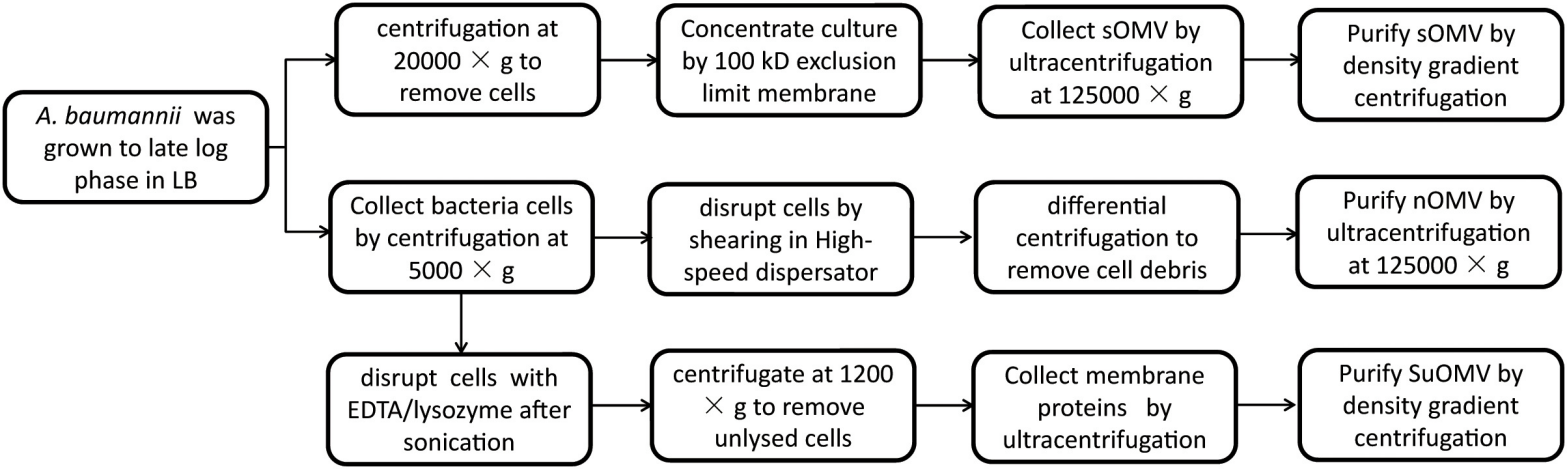
Flow chart for the preparation of preparation of different AbOMVs.

**Figure 2:**
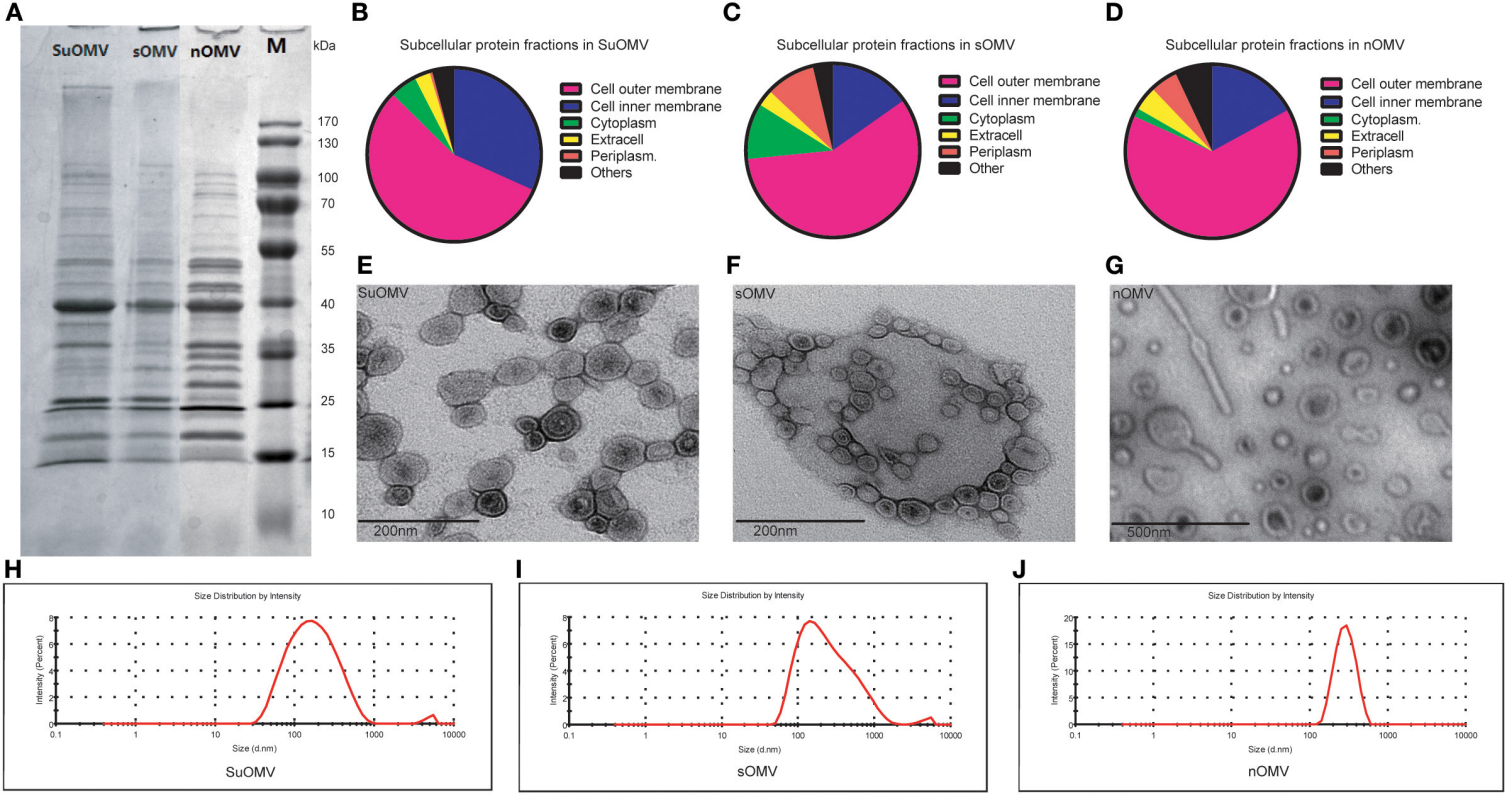
The characterization of the three AbOMVs. sOMV was extracted from the concentrated cell culture supernatant, and SuOMV and nOMV were prepared from the bacterial cells. **(A)** The SDS-PAGE analysis of the AbOMVs. **(B–D)** The predicted subcellular protein fractions of the three AbOMVs. The TEM **(E-G)** and size distribution **(H–J)** of AbOMVs.

The procedures involved in the preparation of different AbOMVs were shown in Figure 1. After purification, 0.129 mg protein per gram of biomass (wet weight) was obtained in sOMV, 0.677 mg protein/g biomass in nOMV, and 3.382 mg protein/g biomass in SuOMV (Table 1). The protein patterns of AbOMVs prepared from different methods were firstly determined by SDS-PAGE. As shown in Figure 2A, the three AbOMVs were similar but not identical in protein patterns. Total proteins in AbOMV were digested with trypsin and the compositions were further analyzed using a LTQ-Orbitrap Velos Pro MS spectrometer (Tables S1–S3). Two hundrend forty-six proteins were identified (>1 unique peptide identified for each protein) in sOMV, 454 proteins in SuOMV, and 397 proteins in nOMV. The most abundant protein in all three AbOMVs was outer membrane protein A (OmpA), representing 33.14% of total protein in sOMV, 28.10% in SuOMV, and 14.15% in nOMV. By predicting the subcellular localization of all proteins in the three AbOMVs using a web-based online tool (Psort, https://www.psort.org/), some significant differences were shown in their subcellular protein fractions, though outer membrane proteins account for the highest proportion of total proteins in all of them. As shown in Figures 2B–D, among the AbOMVs, SuOMV contains the highest percentage of inner membrane proteins, while sOMV contains the most periplasm proteins.

**Table 1:** Protein yield of AbOMVs from three extraction processes.

**AbOMVs**	**Liquid culture (L)**	**Wet weight (g)**	**Total protein (mg)**	**Protein yield (mg/g wet weight)**
sOMV	10	30	3.860	0.128
nOMV	10	30	20.300	0.677
SuOMV	5	10	33.802	3.382

The morphology of purified AbOMVs was therefore determined by TEM, as shown in Figures 2E–G: the three AbOMVs were spherical, double membrane structures. sOMV had an average diameter of 183.3 nm with a polydispersity index (PdI) of 0.364. Compared with sOMV, SuOMV had a smaller average diameter of 142.9 nm with PdI of 0.285, while nOMV had a larger size of 269.9 nm with PdI of 0.086 (Figures 2H–J). SuOMV, sOMV, and nOMV had the average zeta potential value of −8.69, −9.64, and −12.60 mV, respectively. As shown in Table 2, LPS were detected in all three AbOMVs, and nOMV had the highest level of LPS content (0.417 nmol/μg). Only trace amounts of residual DNA was detected in SuOMV (0.4 ng/μg). Together, the three AbOMVs from different preparation processes showed distinct performances in morphology, protein composition, and residues.

**Table 2:** LPS and DNA content of AbOMVs from three extraction processes.

**Residue**	**sOMV**	**nOMV**	**SuOMV**
LPS (nmol/μg)	0.139	0.417	0.103
DNA (ng/μg)	ND	ND	0.4

*ND, not detected*.

### AbOMVs Promoted BMDCs Maturation

**Figure 3:**
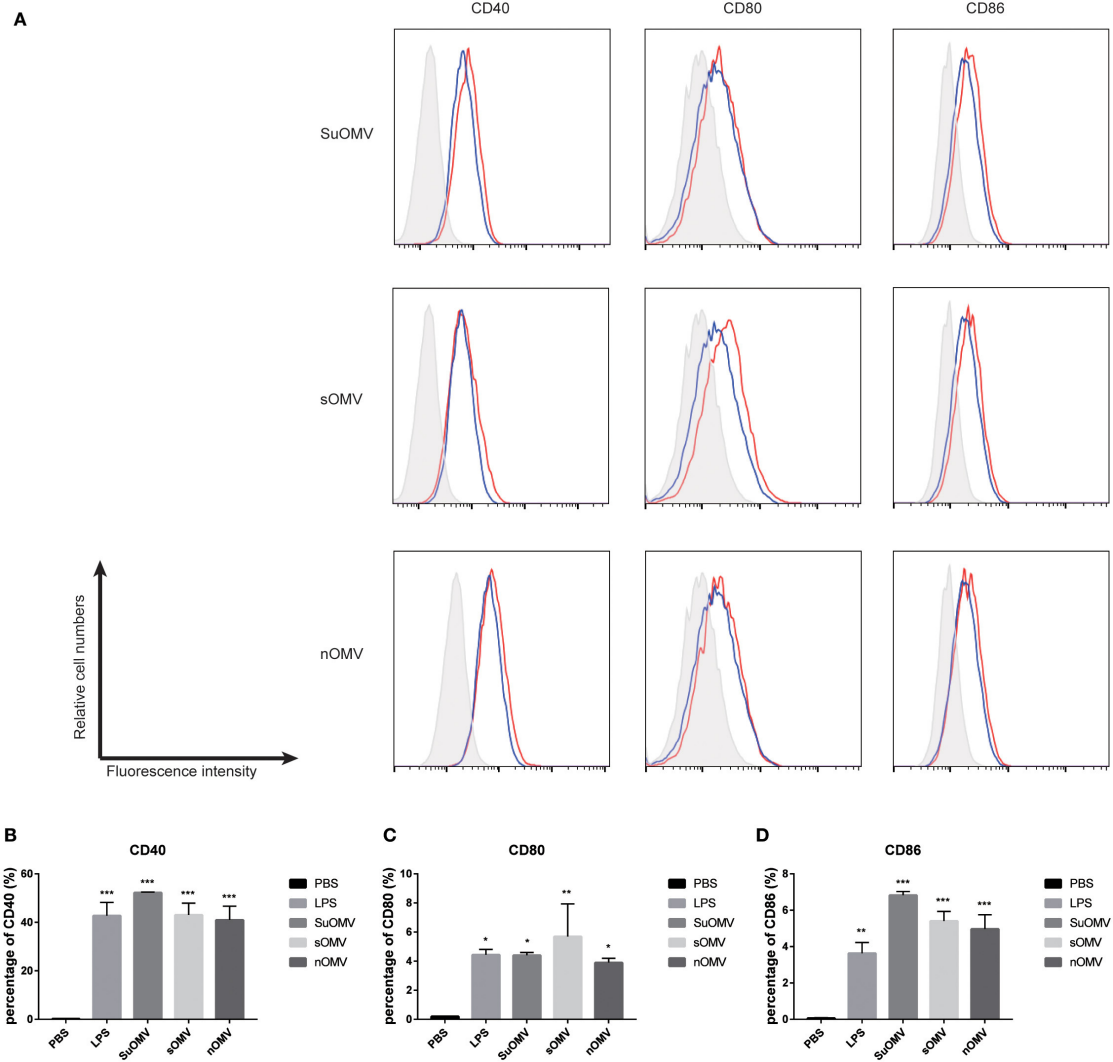
The expression of maturation surface markers in BMDCs treated with the AbOMVs. BMDCs were incubated with PBS, LPS, SuOMV, sOMV, or nOMV for 48 h and analyzed for surface expression of CD40, CD80, and CD86. **(A)** A representative set of flow cytometry histograms. Black line: cells treated with PBS. Blue line: cells treated with LPS. **(B–D)** Normalized expression level of maturation markers. Data are expressed as mean ± S.D., *n* = 3. *P < 0.05, **P < 0.01, ***P < 0.001 compared with PBS control.

To compare the maturation efficacy of dendritic cells by the three AbOMVs, BMDCs were stimulated with PBS, SuOMV, sOMV, nOMV, or LPS for 48 h. FACS results revealed that a significant increased expression of costimulatory molecules (CD40, CD80, and CD86) was elicited by the three AbOMVs (Figures 3A–D). Though nOMV had the highest level of LPS, SuOMV showed the most potent stimulating effect among the three AbOMVs, indicating that DCs maturation induced by AbOMVs was not only attributed to the residual LPS (38).

### AbOMVs Enhanced BMDCs Cytokines Secretion

**Figure 4:**
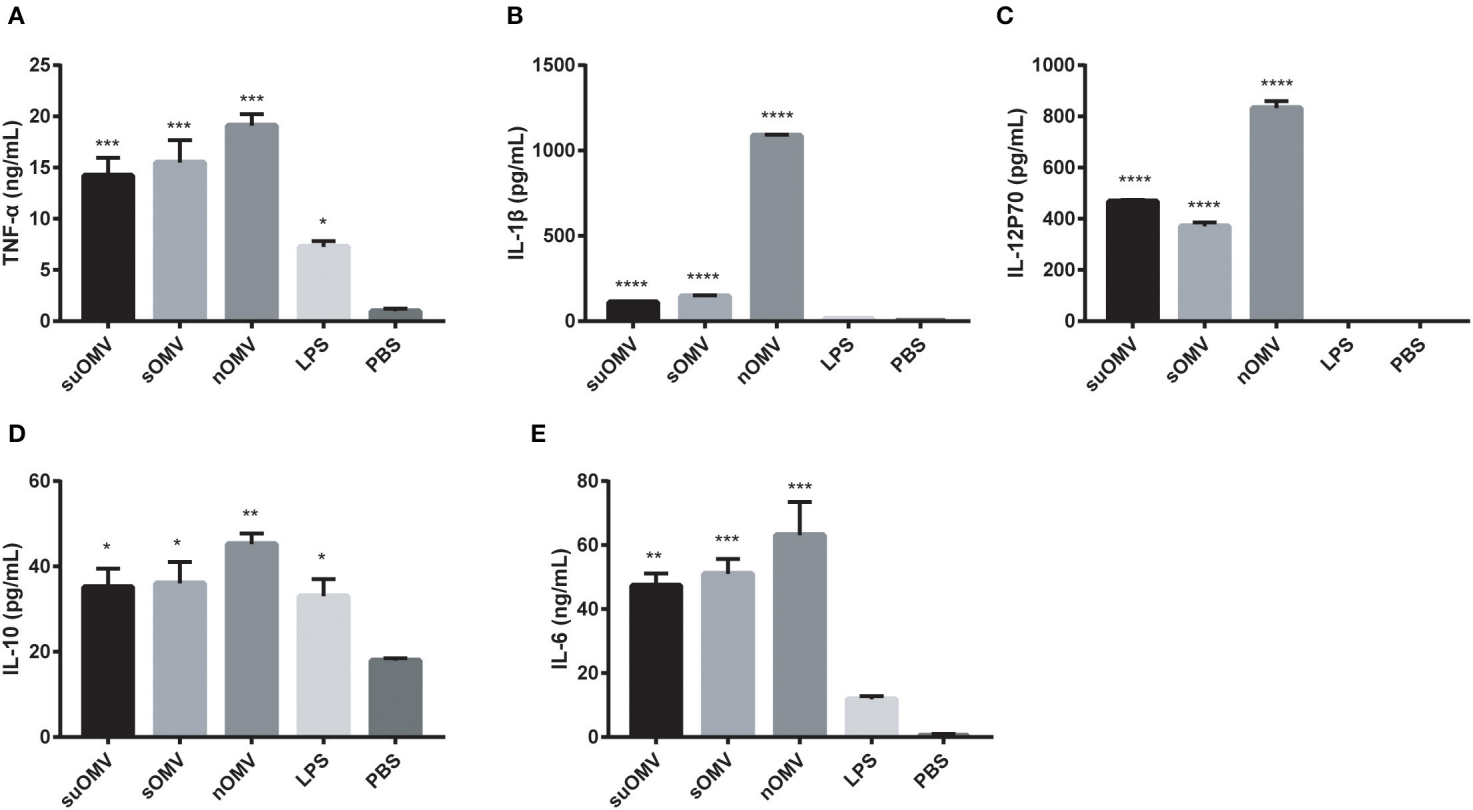
Cytokines expression in the supernatants of BMDCs stimulated with the AbOMVs. BMDCs were stimulated with PBS, LPS, SuOMV, sOMV, or nOMV for 48h. ELISA assays were used to measure the accumulation of TNF-α **(A)**, IL-1β **(B)**, IL-12p70 **(C)**, IL-10 **(D)**, and IL-6 **(E)** in the supernatants of culture. Data are expressed as mean ± S.D., *n* = 3. *P < 0.05, **P < 0.01, ***P < 0.001 compared with PBS control.

To determine whether AbOMVs could induce BMDCs cytokines secretion, the levels of cytokines in the supernatants of BMDCs stimulated with AbOMVs were measured by ELISA. As shown in Figures 4A,B, treatment with AbOMVs resulted in the increased production of pro-inflammatory cytokines TNF-α and IL-1β. Significant secretion of Th1, Th2, and Th17 polarizing immune cytokines IL-12p70, IL-10, and IL-6 was also observed (Figures 4C–E), which suggested that AbOMVs had the potential to activate the innate immune response and T-cell mediated response (39).

### Protective Effect of the AbOMV Varied by Preparation Method and Immunization Route

**Figure 5:**
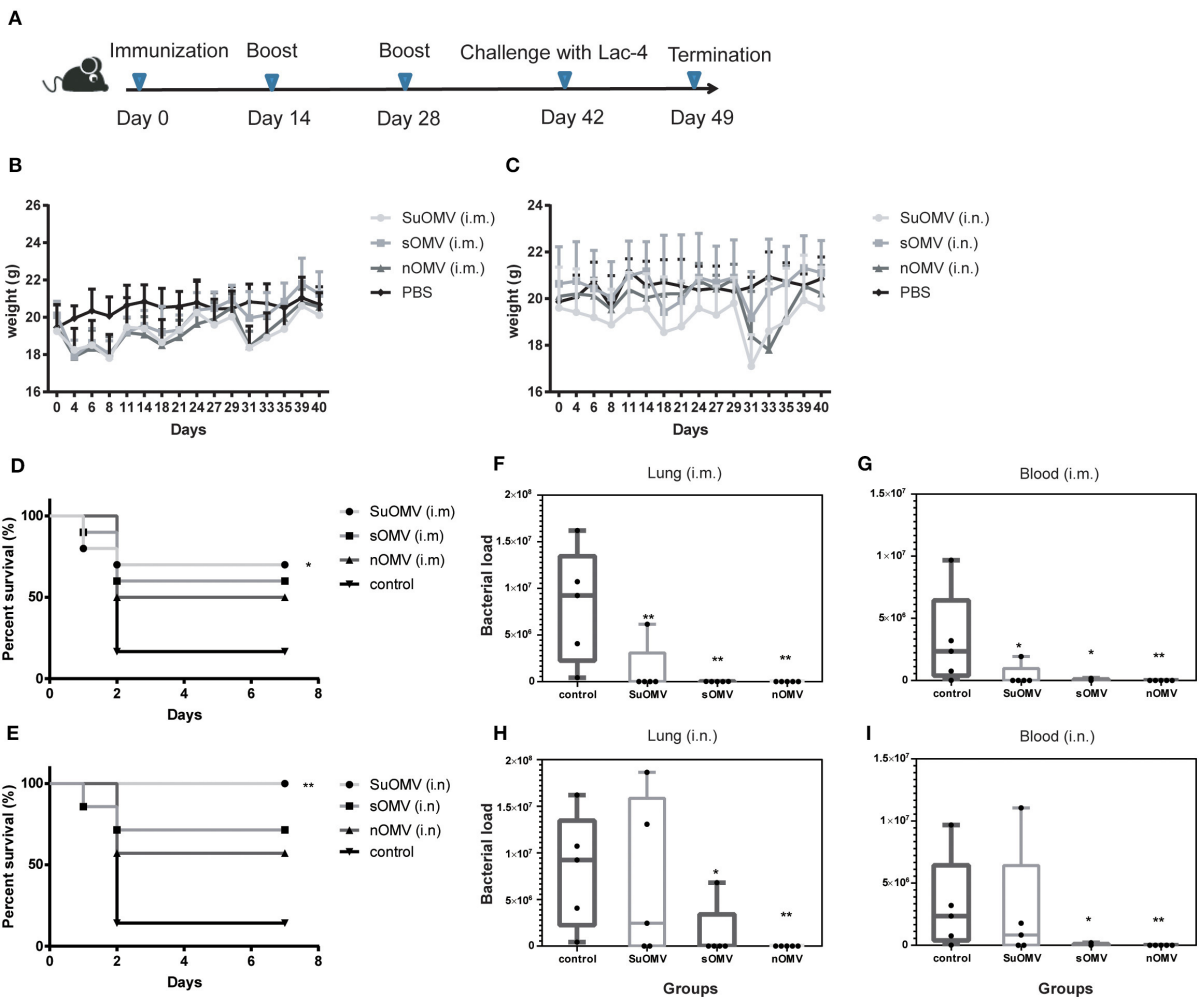
Protective effect of AbOMVs against lethal and sub-lethal *A. baumannii* challenge. **(A)** Timeline representation of immunization schedule and experimental procedures. BALB/c mice were intramuscularly or intranasally immunized with either of the AbOMVs. Controls were treated with PBS. Two weeks after the final vaccination, mice were challenged intratracheally with *A. baumannii* Lac-4. **(B,C)** Weight changes in mice over the duration of vaccination were recorded for preliminary safety evaluation. **(D,E)** 7-days survival rates of immunized mice after lethal challenge were calculated (*n* = 10). Bacterial loads in lungs **(F,H)** and blood **(G,I)** of immunized mice 24 h post sub-lethal i nfection were determined by CFU counts of serial 10-fold dilutions on TSA. Dots show values in each mouse, boxes show the range for each group (min, median, and max), *n* = 5. *P < 0.05, **P < 0.01, compared with PBS control.

To assess the preliminary safety and the protective potential of the three AbOMVs, C57BL/6 mice were intramuscularly immunized with each AbOMV absorbed to alum adjuvant or intranasally immunized with each AbOMV alone (Figure 5A), and the body weight changes were measured (Figures 5B,C). Although weight loss was observed in both the intramuscularly and intranasally vaccinated groups, the mice body weight generally returned to a normal level 2 weeks after the final immunization (day 42). At day 42, immunized and control mice were intratracheally challenged with *A. baumannii* LAC-4, and the survival rates were observed until 7 days after challenge. 80% of mice in the control group died in the first 2 days post challenge, and the final 7-days survival rates of SuOMV, sOMV, and nOMV were measured at 70, 60, and 50% in the intramuscular immunization groups (Figure 5D). Higher 7-days survival rates were obtained in intranasally vaccinated groups compared with those intramuscularly vaccinated with the same antigen, and SuOMV conferred the highest survival rates of 100% (Figure 5E). In addition, bacterial burdens in the lung and blood at 24 h after sub-lethal challenge were determined. As shown in Figures 5F,I, vaccination with either type of AbOMV resulted in clearance of the *A. baumannii* challenge inoculum from the lung and blood, and no bacteria were observed in majority of the vaccinated mice. However, all mice in PBS group harbored *A. baumannii* in the lung and blood at this time-point. Those results indicated that the AbOMVs prepared by different methods were able to provide protection against lethal and sublethal *A. baumannii* challenge but varied by their preparation method and immunization route.

### Different Levels of Specific IgG and IgG Subclasses Response Were Induced by the Three AbOMVs

**Figure 6:**
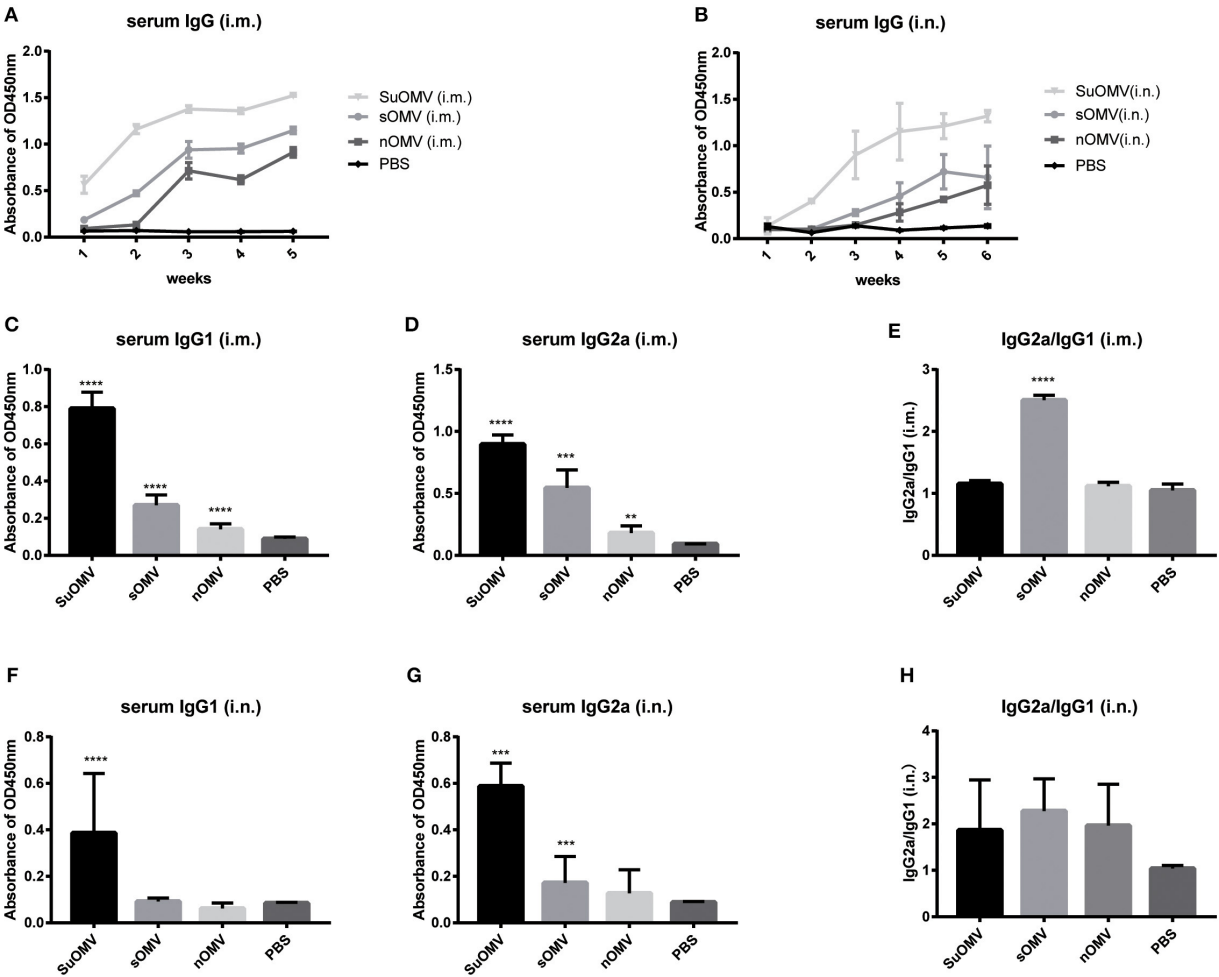
Specific IgG and IgG subclasses profiles of mice immunized with either of the AbOMVs. BALB/c mice were immunized intramuscularly or intranasally with SuOMV, sOMV, or nOMV. The serum was collected every week until the time of challenge. **(A,B)** Specific serum IgG antibody titers were measured by ELISA. **(C,D,F,G)** The levels of specific IgG1 and IgG2a in serum samples were tested. **(E,H)** The IgG2a/IgG1 ratio (ratios>1 and <1 indicate a Th1 and Th2 polarized response, respectively). Data are expressed as mean ± S.D., *n* = 10. **P < 0.01, ***P < 0.001, ****P < 0.0001, compared with PBS control.

In order to investigate the protective mechanism of AbOMVs against *A. baumannii* infection, the levels of specific serum IgG antibody of immunized mice were determined by ELISA. As shown in Figures 6A,B, both intramuscular and intranasal immunization with AbOMVs elicited significant serum specific IgG antibody response compared with PBS group. Through both immunization routes, the order of potency to induce specific IgG response, from most to least potent, was SuOMV, sOMV, nOMV, which was consistent with their protective efficacy. Moreover, intramuscular immunization generally induced more robust IgG level than the same AbOMV given intranasally. IgG1 and IgG2a as markers for Th2 and Th1 responses, respectively (40), and AbOMV-specific IgG1 and IgG2a levels, were also determined. As shown in Figures 6C,D, through intramuscular injection, both SuOMV and sOMV induced significant IgG1 and IgG2a responses, and the highest IgG1 and IgG2a levels were observed in the SuOMV group. However, there were no significant difference in response levels between the nOMV group and PBS group. In addition, though SuOMV and sOMV induced a significant IgG2a response through intranasal immunization (Figures 6F,G), only SuOMV significantly enhanced the expression level of specific IgG1. The ratio of IgG2a to IgG1 (IgG2a/IgG1) in the sOMV group was significantly higher than the PBS and other immune groups, with the ratio >1, suggesting that sOMV induced an antigen-specific Th1-biased response (Figure 6E). In all the intranasal immunization groups, IgG2a/IgG1 ratio was >1, indicating that a specific Th1-biased response was elicited when mice were intranasally immunized with the AbOMVs (Figure 6H).

### Intranasal Immunization With the AbOMVs Elevated Specific sIgA Production in Mucosal Sites

**Figure 7:**
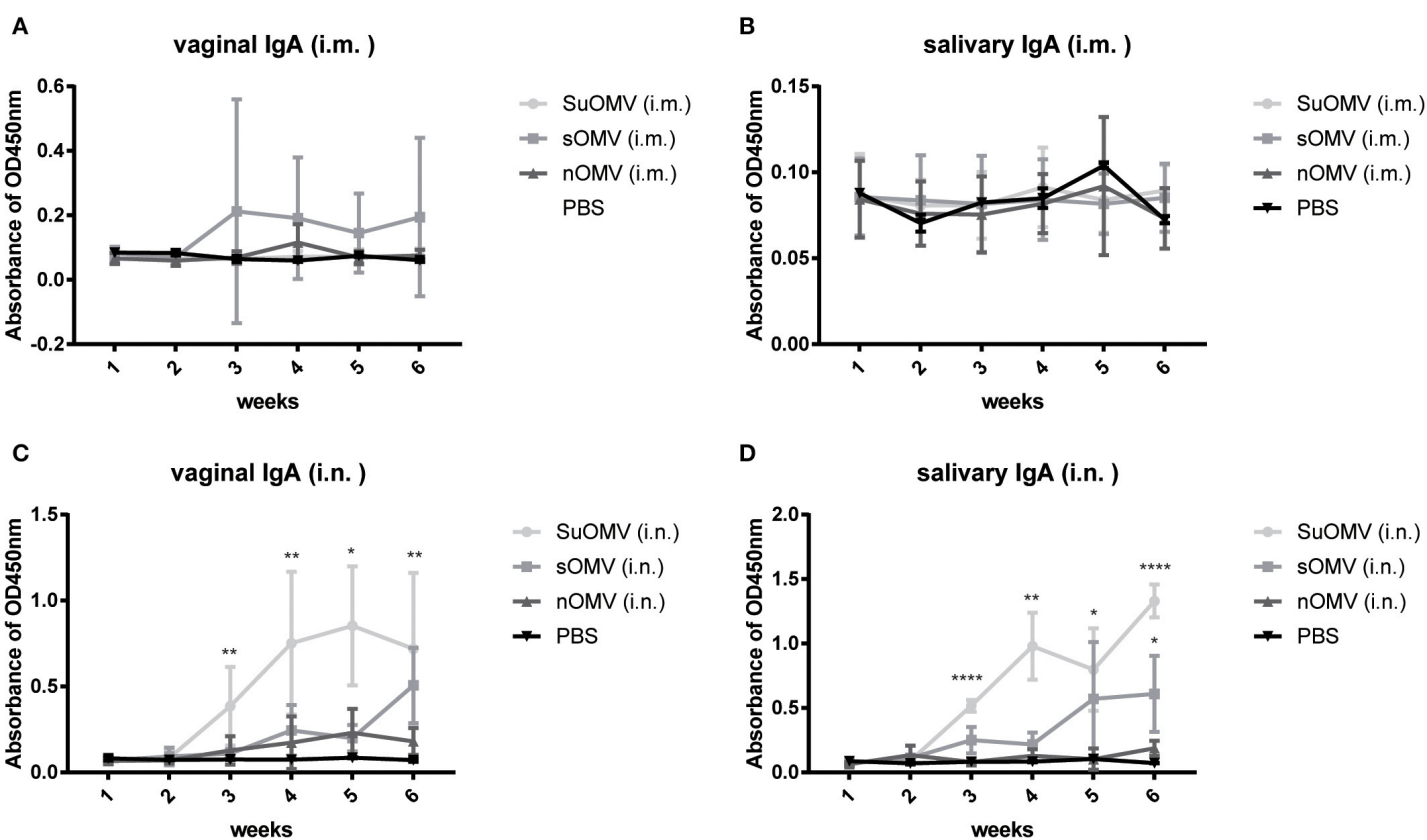
Specific sIgA responses elicited by immunization with either of the AbOMVs. BALB/c mice were immunized intramuscularly or intranasally with SuOMV, sOMV, or nOMV. Saliva and vaginal wash were collected every week until the time of challenge. Secretory IgA levels in vaginal wash **(A,C)** and saliva **(B,D)** were measured by ELISA. Data are expressed as mean ± S.D., *n* = 10. *P < 0.05, **P < 0.01, ****P < 0.0001, compared with PBS control.

Considering that mucosal secretary IgA (sIgA) antibody played a role in blocking bacterial infection (41), secretions (saliva and vaginal wash) were collected to detect the level of sIgA. As shown in Figures 7A,B, intramuscular immunization with AbOMVs failed to induce significantly elevated salivary or vaginal sIgA responses, though mice in the SuOMV group showed a trend toward higher sIgA levels. However, compared with other treatments, more robust mucosal sIgA in secretions were elicited in groups intranasally immunized with sOMV or SuOMV, and SuOMV triggered the highest mucosal sIgA production among them (Figures 7C,D). Those results indicated that intranasal administration with AbOMVs were superior to intramuscular administration for inducing stronger antigen-specific sIgA antibody responses, which may be related to their significantly better protective role against *A. baumannii* infection.

## Discussion

OMVs are spherical nano-sized proteoliposomes shed from the outer membrane of Gram-negative bacteria, containing components capable of stimulating humoral and cellular immune responses, which have gained interest for use as non-replicating vaccines. The licensed MenB OMV vaccines induce a protective immune response and have been proven effective in controlling local epidemic outbreaks. OMV vaccines had also been developed to combat infectious diseases caused by other pathogens in animal models, such as *Helicobacter pylori* (42), *Pseudomonas aeruginosa* (43), *Escherichia coli* (44), and *Streptococcus pneumonia* (45), which offered attractive strategies for protection against bacterial infection.

*A. baumannii* is becoming a common global concern due to the emergence of multi-drug (MD) or pan-drug (PD) resistant strains in the population. Confronting the issue of antimicrobial resistance by developing vaccines against the resistant pathogen is becoming a common strategy AbOMVs are a potential vaccine candidate which have been identified against *A. baumannii* infection. In the development of meningococcal OMV, it was found that MenB OMV obtained by different preparation methods exhibited significant differences in morphology, protein composition, LPS residue, and even SBA (23, 27), which triggered our interest to investigate whether the preparation method of AbOMVs would also affect their protective efficacy against *A. baumannii* in a mice model.

In this study, three different methods were used to prepare AbOMVs. sOMV was obtained from the concentrated culture supernatant, and additional density gradient centrifugation was carried out for further purification, compared with the methods described previously (27). SuOMV and nOMV were formed by the reassembly of outer membrane components after disruption of the cell membrane integrity. To obtain SuOMVs, *A. baumannii* cells were firstly disrupted by EDTA/lysozyme treatment after sonication, and then the SuOMVs were separated from the total protein by sucrose gradient isopycnic centrifugation. nOMVs were also obtained from cells, but the cells were briefly sheared with a high-speed dispersator, and nOMVs were collected and purified by differential centrifugation. The highest yield was obtained in SuOMV preparation, almost 26 times that of sOMV. Although the preparation methods were different, the three AbOMVs had some common aspects, including that they were spherical and had double membrane structures, outer membrane proteins accounted for the highest proportion of total proteins, and the protein with the highest content was OmpA.

However, more differences were observed among the three AbOMVs. The particle size of sOMV and suOMV were similar (<200 nm), which was significantly smaller than that of nOMV (>200 nm), but the aggregates in the two samples were more polydisperse (PDI ≈ 0.3) (46). In addition to outer membrane proteins, subcellular protein fractions in the three AbOMVs were significantly different. sOMV was naturally secreted by *A. baumannii*, always composed of high percentage of periplasm proteins (47), which have been addressed in other studies. Moreover, due to the fact that inner membrane proteins were also peeled away during cells treated by EDTA/lysozyme, the highest fractions of inner membrane proteins were also included in the SuOMV. Compared with nOMV, lower levels of LPS were detected in sOMV and SuOMV, which may have benefited from the final purification with density gradient centrifugation.

DCs are the most potent antigen-presenting cells (APCs) involved in bridging innate and adaptive immunity (48). Upon maturation, DCs are capable of priming naïve T cells and initiating primary T cell-mediated immune responses (49). In the current study, the three AbOMVs treatment significantly promoted BMDCs maturation, which was characterized by the up-regulation of costimulatory molecules CD40, CD80, and CD86. In addition, the productions of TNF-α, IL-1β, IL-12p70, IL-10, and IL-6 in the supernatants of BMDCs were also significantly elevated, suggesting that BMDCs stimulated by the AbOMVs had the potential to activate T cell-mediated immune responses. LPS in AbOMVs may play an important role in BMDCs maturation, which had been proven to be desirable for its high adjuvant activity in MenB OMV vaccine (50). Among the three AbOMVs, though nOMV had the highest level of residual LPS, SuOMV showed the most potent stimulating effect, indicating that other mechanisms may contribute to the BMDCs activation and maturation.

Vaccination with the three AbOMVs via both routes conferred significant protection against lethal and sub-lethal *A. baumannii* challenge. SuOMV provided the highest level of protective efficacy against lethal infection. However, lower reduction of bacterial burdens was observed in the SuOMV group 24 h post sublethal challenge. To further confirm the protective effect of the three AbOMVs, the bactericidal activity of antibodies induced by them was evaluated. The highest level of serum bactericidal titers was observed in mice immunized with SuOMV, whether intramuscular or intranasal immunization, which support the result of the survival rate in the lethal model (data not shown). We speculated that 24 h post challenge may not be the most appropriate investigated time-point.

The mechanisms of vaccine-mediated protection against *A. baumannii* infection were not fully understood. In our study, SuOMV showed the most potency to stimulate BMDCs maturation. All three AbOMVs were able to induce specific serum IgG responses, whether through intramuscular or intranasal immunization. However, only intranasal immunization with AbOMVs promoted specific sIgA production in mucosal secretions, which may be a possible reason why intranasal vaccination conferred more potent protective efficacy, though higher levels of serum IgG were induced by intramuscular immunization. Earlier studies suggested that *A. baumannii* vaccine dose escalation resulted in an enhanced Type 2 immune response (51). However, a Th1-biased response was elicited by intranasal vaccination, characterized by higher levels of IgG2a and lower levels of IgG1 in the serum (IgG2a/IgG1 >1), which may also contribute to the better protective effect in those groups (52). By two routes of administration, SuOMV induced the highest level of specific IgG or mucosal sIgA among the three AbOMVs, which was consistent with the fact that SuOMV provided the best protection against *A. baumannii* lethal infection.

Three AbOMVs exhibited significant differences in particle size, surface charge, and protein compositions, which may influence their protective efficacy. Small particles are always more efficiently internalized by APCs than larger particles (53, 54). Moreover, size is an important factor that influences the entry of antigens to the lymphatic system (55). Particles of 20–200 nm efficiently enter the lymphatic system (56), while particles larger than 200 nm need to be carried into the lymphatic system by specialized cells, such as DCs (57). In the current study, SuOMV was the smallest in particle size (142.9 nm), while SuOMV and sOMV had larger sizes with an average diameter of 183.3 and 269.9 nm. Among three AbOMVs, SuOMV may be internalized by APCs most efficiently. Also, SuOMV and sOMV may enter the lymphatic system more efficiently than nOMV. Particle surface charge is also believed to have an effect on vaccine-induced immune responses (58). Neutral or positive charges may be favorable in triggering specific immune responses (59, 60). SuOMV, sOMV, and nOMV had different zeta potential, which may also be associated with their potency to induce different levels of immune response. OmpA was found to be a predominant virulence factor which plays key roles in regulating the adhesion, aggressiveness, and biofilm formation of *A. baumannii* (61, 62). The highest level of OmpA-specific IgG was observed in mice vaccinated with SuOMV, though the highest percentage of OmpA was included in sOMV. Consistent with the fact that nOMV contained the minimum percentage of OmpA, nOMV induced the lowest level of OmpA-specific IgG response (data not shown). Moreover, SuOMV contained more proteins compared with nOMV and sOMV, which may also be the reason that SuOMV provided the most potent protective effect.

The inclusion of LPS into OMV was always inevitable, because LPS was required for OMV formation (63). Though LPS had been suggested as an alternative adjuvant for OMV vaccines, LPS was also the main factor affecting the safety (23). In the preliminary experiments, sodium deoxycholate was used to prepare AbOMV in order to reduce LPS content, but vesicles were not found by TEM. Weight changes of mice after immunization indicated that AbOMVs had an acceptable level of safety. In the future, OMV vaccines against *A. baumannii* based on nontoxic LPS mutants will be developed (64). A recent study demonstrated that different types of MVs other than OMVs can be formed by Gram-negative bacteria (65); other types of MVs will be extracted and their protective effect will be evaluated in our future study. In addition to density gradient centrifugation, detergents were screened to further reduce the content of LPS in SuOMV and nOMV. In addition, shear speed will be studied to prepare nOMV in different particle sizes, and the relationship of particle size and the protective efficacy will be further determined. The detailed mechanisms of immune stimulation by the three AbOMVs would be the object of intensive study.

## Conclusions

In the current study, two novel methods were established to prepare AbOMVs (SuOMV and nOMV) from *A. baumannii* cells, by which a significantly higher yield was obtained compared with isolation from the culture supernatant (sOMV). In addition, three AbOMVs exhibited significant differences in particle size, surface charge, protein composition, and LPS/DNA content. Among three AbOMVs, SuOMV provided the most potent protective efficacy against *A. baumannii* challenge, and intranasal vaccination with the AbOMV provided more robust protection compared with intramuscular vaccination. Finally, both serum IgG response and mucosal sIgA response may contribute to the protection of AbOMVs vaccine.

## Data Availability Statement

All datasets generated for this study are included in the article/Supplementary Material.

## Ethics Statement

The animal study was reviewed and approved by Animal Ethical and Experimental Committee of the Third Military Medical University.

## Author Contributions

H-BL and Q-MZ designed the experiments and wrote the manuscript. SL, D-QC, LJ, SS, ZJ, Z-LJ, H-WS, HZ, W-JZ, D-SL, and PL carried out the experiments. H-BL, SL, A-NZ, and LJ analyzed experimental results. All authors contributed to the article and approved the submitted version.

## Conflict of Interest

The authors declare that the research was conducted in the absence of any commercial or financial relationships that could be construed as a potential conflict of interest.
