# Supplementary material for: Development of Different Methods for Preparing Acinetobacter baumannii Outer Membrane Vesicles Vaccine: Impact of Preparation Method on Protective Efficacy
Source: Front Immunol. 2020 Jun 23;11:1069. doi: 10.3389/fimmu.2020.01069 (PMC7324643; doi:10.3389/fimmu.2020.01069)
Supplement: Supplementary file 1 [file Data_Sheet_1.docx]

**Table S1 The 50 most abundant proteins identified by LTQ-Orbitrap MS Spectrometry in the suOMV**

| Protein | Acc.No | MW [kDa] | | Coverage | Area | Subcellular location |
| --- | --- | --- | --- | --- | --- | --- |
| outer membrane protein A | ABO13246 | | 38.4 | 58.99 | 6.783E9 | Cell outer membrane |
| putative outer membrane protein | ABO11316 | | 22.5 | 59.45 | 1.360E9 | Cell outer membrane |
| hypothetical protein | ABS90051 | | 11.2 | 78.07 | 7.245E8 | Cell outer membrane |
| bacteriolytic lipoprotein entericidin B | ABO13215 | | 4.9 | 41.30 | 6.886E8 | Others |
| membrane-bound ATP synthase F0 sector, subunit b | ABO10634 | | 17.0 | 58.33 | 6.570E8 | Cell inner membrane |
| putative signal peptide | ABO11459 | | 41.2 | 74.80 | 5.274E8 | Cell inner membran |
| putative lipoprotein | ABO11441 | | 12.9 | 28.81 | 5.129E8 | Cell outer membrane |
| cytochrome o ubiquinol oxidase subunit II | ABO12593 | | 38.9 | 40.29 | 4.439E8 | Cell inner membrane |
| putative hemolysin | ABO11749 | | 15.7 | 60.42 | 3.875E8 | Cell inner membrane |
| hypothetical protein | ABS89968 | | 14.3 | 10.07 | 3.835E8 | Fimbrium |
| cytochrome o ubiquinol oxidase subunit I | ABO12594 | | 74.5 | 15.69 | 3.507E8 | Cell inner membrane |
| succinate dehydrogenase flavoprotein subunit | ABO13126 | | 67.0 | 65.47 | 2.902E8 | Cytoplasm |
| cytochrome d terminal oxidase polypeptide subunit I | ABO12351 | | 58.6 | 29.92 | 2.772E8 | Cell inner membrane |
| peptidoglycan-associated lipoprotein precursor | ABO13012 | | 20.1 | 74.87 | 2.734E8 | Cell outer membrane |
| putative membrane protein | ABO11211 | | 59.1 | 17.38 | 2.670E8 | Cell inner membrane |
| OmpA/MotB | ABO11623 | | 48.9 | 58.39 | 2.388E8 | Cell inner membrane |
| succinate dehydrogenase iron-sulfur subunit | ABO13127 | | 26.8 | 44.07 | 2.204E8 | Cell inner membrane |
| hypothetical protein | ABO11890 | | 35.0 | 63.58 | 2.183E8 | Cell inner membrane |
| membrane-bound ATP synthase F0 sector, subunit c | ABO10633 | | 8.4 | 22.22 | 2.128E8 | Cell inner membrane |
| cytochrome d terminal oxidase polypeptide subunit II | ABO12352 | | 41.5 | 15.49 | 2.101E8 | Cell inner membrane |
| hypothetical protein | ABO12306 | | 17.0 | 38.71 | 2.050E8 | Cell outer membrane |
| putative signal peptide | ABO12682 | | 13.3 | 61.11 | 1.922E8 | Cell outer membrane |
| malate dehydrogenase FAD/NAD(P)-binding domain | ABO11355 | | 60.4 | 56.59 | 1.831E8 | Cytoplasm |
| toluene tolerance efflux transporter | ABO13498 | | 24.1 | 69.03 | 1.765E8 | Cell inner membrane |
| putative membrane protein | ABO12001 | | 13.6 | 23.97 | 1.728E8 | Cell inner membrane |
| putative protein (DcaP-like) | ABO13166 | | 47.3 | 12.44 | 1.600E8 | Extracell |
| FKBP-type peptidyl-prolyl cis-trans isomerase (rotamase) | ABO10543 | | 25.9 | 58.33 | 1.595E8 | Cell outer membrane |
| glucose dehydrogenase | ABO13254 | | 28.2 | 28.35 | 1.592E8 | Cell inner membrane |
| putative signal peptide | ABO13577 | | 21.7 | 73.47 | 1.527E8 | Cell inner membrane |
| mechanosensitive channel | ABO13240 | | 15.8 | 31.47 | 1.412E8 | Cell inner membrane |
| hypothetical protein | ABO12359 | | 13.9 | 43.70 | 1.189E8 | Cytoplasm |
| putative membrane protease subunit | ABO13171 | | 31.0 | 54.23 | 1.169E8 | Cell inner membrane |
| putative preprotein translocase IISP family membrane subunit | ABO13318 | | 11.7 | 24.77 | 1.158E8 | Cell inner membrane |
| glucose dehydrogenase | ABO13253 | | 44.3 | 40.44 | 1.089E8 | Cell inner membrane |
| hypothetical protein | ABO10958 | | 41.1 | 38.58 | 1.007E8 | Cell outer membrane |
| EsvG | ABO10837 | | 40.1 | 46.85 | 9.674E7 | Cytoplasm |
| putative competence protein (ComL) | ABO11274 | | 42.8 | 61.30 | 9.065E7 | Cell inner membrane |
| Cu/Zn superoxide dismutase | ABO13540 | | 19.3 | 69.11 | 8.866E7 | Periplasm |
| D-amino acid dehydrogenase small subunit | ABO12411 | | 46.5 | 30.43 | 8.782E7 | Cytoplasm |
| hypothetical protein | ABO13405 | | 24.9 | 36.24 | 8.457E7 | Extracell |
| cell division protein | ABO13094 | | 69.6 | 36.61 | 8.318E7 | Cytoplasm |
| putative lipoprotein precursor | ABO13390 | | 18.0 | 45.28 | 8.230E7 | Cell inner membrane |
| membrane-bound ATP synthase F1 sector, alpha-subunit | ABO10636 | | 55.4 | 33.27 | 8.081E7 | Cell inner membrane |
| D-ala-D-ala-carboxypeptidase; penicillin-binding protein 5 (precursor) | ABO12853 | | 41.8 | 24.87 | 7.891E7 | Cell inner membrane |
| hypothetical protein | ABS90169 | | 17.5 | 45.06 | 7.813E7 | Cell inner membrane |
| protein tyrosine kinase | ABO10544 | | 81.4 | 44.09 | 7.786E7 | Cell inner membrane |
| general secretion pathway protein G | ABO10830 | | 20.9 | 30.57 | 7.720E7 | Cell inner membrane |
| Inner membrane protein (IMP) integration factor | ABO13384 | | 40.5 | 21.04 | 7.668E7 | Cell inner membrane |
| hypothetical protein | ABS90044 | | 18.0 | 31.93 | 7.489E7 | Fimbrium |
| hypothetical protein | ABO10957 | | 25.8 | 43.97 | 7.390E7 | Cell inner membrane |

**Table S2. The 50 most abundant proteins identified by LTQ-Orbitrap MS Spectrometry in the sOMV**

| Protein | Acc.No | MW [kDa] | Coverage | Area | Subcellular location |
| --- | --- | --- | --- | --- | --- |
| outer membrane protein A | ABO13246 | 38.4 | 42.98 | 5.740E9 | Cell outer membrane |
| DNA strand exchange and recombination protein | ABO12389 | 37.9 | 6.88 | 1.127E9 | Cytoplasm |
| putative outer membrane protein | ABO11316 | 22.5 | 46.54 | 1.113E9 | Cell outer membrane |
| putative signal peptide | ABO12391 | 32.5 | 80.61 | 1.020E9 | Cell inner membrane |
| tolerance to colicins E2 E, A, and K | ABO13011 | 46.5 | 62.21 | 9.778E8 | Periplasm |
| peptidoglycan-associated lipoprotein precursor | ABO13012 | 20.1 | 77.01 | 6.425E8 | Cell outer membrane |
| bacteriolytic lipoprotein entericidin B | ABO13215 | 4.9 | 52.17 | 5.541E8 | Others |
| putative signal peptide | ABO13088 | 15.5 | 30.00 | 4.377E8 | Cell outer membrane |
| putative hemolysin | ABO11749 | 15.7 | 65.97 | 4.096E8 | Cell inner membrane |
| lipoprotein precursor | ABO12850 | 29.6 | 49.28 | 3.160E8 | Cell outer membrane |
| acyl coenzyme A reductase | ABO13615 | 32.7 | 7.43 | 2.821E8 | Cytoplasm |
| putative signal peptide | ABO11459 | 41.2 | 61.25 | 2.524E8 | Cell inner membrane |
| putative lipoprotein precursor | ABO13390 | 18.0 | 52.83 | 2.192E8 | Cell inner membrane |
| hypothetical protein | ABO12306 | 17.0 | 47.10 | 1.927E8 | Cell outer membrane |
| putative signal peptide | ABO12682 | 13.3 | 55.56 | 1.867E8 | Cell outer membrane |
| putative protein (DcaP-like) | ABO13166 | 47.3 | 12.44 | 1.858E8 | Extracell |
| membrane-bound lytic murein transglycosylase B | ABO12738 | 36.8 | 33.93 | 1.419E8 | Cell outer membrane |
| putative Zn-dependent protease with chaperone function | ABO11610 | 27.6 | 42.15 | 1.378E8 | Cell inner membrane |
| putative signal peptide | ABO12852 | 15.4 | 55.71 | 1.125E8 | Cytoplasm |
| putative signal peptide | ABO11482 | 24.8 | 71.91 | 1.029E8 | Periplasm |
| hypothetical protein | ABO12767 | 51.4 | 44.12 | 1.022E8 | Extracell |
| Cu/Zn superoxide dismutase | ABO13540 | 19.3 | 53.40 | 9.635E7 | Periplasm |
| putative competence protein (ComL) | ABO11274 | 42.8 | 62.08 | 9.107E7 | Cell inner membrane |
| putative serine protease | ABO12942 | 49.0 | 48.69 | 8.580E7 | Periplasm |
| hypothetical protein | ABO10610 | 13.6 | 39.84 | 7.891E7 | Cell outer membrane |
| hypothetical protein | ABO13757 | 21.0 | 34.76 | 7.275E7 | Periplasm |
| FilF | ABO11139 | 69.8 | 54.12 | 6.568E7 | Extracell |
| putative membrane-bound lytic murein transglycosylase | ABO13365 | 47.0 | 47.21 | 6.311E7 | Cell outer membrane |
| putative signal peptide | ABO13547 | 13.7 | 43.44 | 5.817E7 | Cell outer membrane |
| putative lytic murein transglycosylase soluble | ABO13424 | 73.0 | 47.14 | 5.596E7 | Periplasm |
| hypothetical protein | ABO11890 | 35.0 | 38.98 | 5.511E7 | Cell inner membrane |
| hypothetical protein | ABS90305 | 30.3 | 3.26 | 5.260E7 | Extracell |
| hypothetical protein | ABO13423 | 24.4 | 50.00 | 5.244E7 | Cytoplasm |
| putative toluene tolerance protein (Ttg2D) | ABO13497 | 21.2 | 43.98 | 5.145E7 | Cell inner membrane |
| peptidyl-prolyl cis-trans isomerase | ABO11972 | 49.0 | 54.59 | 5.064E7 | Cell inner membrane |
| hypothetical protein | ABO10958 | 41.1 | 45.14 | 4.864E7 | Cell outer membrane |
| hypothetical protein | ABO12708 | 20.6 | 34.43 | 4.808E7 | Extracell |
| putative peptide signal | ABO13662 | 14.2 | 25.20 | 4.689E7 | Cell outer membrane |
| carboxy-terminal protease | ABO10946 | 80.7 | 42.37 | 4.443E7 | Cell inner membrane |
| ATP-dependent helicase | ABO11636 | 107.6 | 0.96 | 4.214E7 | Cytoplasm |
| hypothetical protein | ABO10874 | 19.3 | 50.30 | 4.077E7 | Cytoplasm |
| hypothetical protein | ABS90126 | 14.5 | 39.10 | 3.881E7 | Cell outer membrane |
| putative outer membrane protein | ABO12396 | 95.5 | 32.75 | 3.572E7 | Cell outer membrane |
| hypothetical protein | ABS90256 | 41.0 | 37.00 | 3.552E7 | Extracell |
| hypothetical protein | ABO11676 | 25.3 | 47.98 | 3.492E7 | Cell inner membrane |
| hypothetical protein | ABO12707 | 88.2 | 14.66 | 3.482E7 | Cell inner membrane |
| putative lipoprotein | ABO11441 | 12.9 | 27.12 | 3.291E7 | Cell outer membrane |
| hypothetical protein | ABS90254 | 27.2 | 26.47 | 3.263E7 | Extracell |
| hypothetical protein | ABS90184 | 13.1 | 31.67 | 3.253E7 | Fimbrium |
| hypothetical protein | ABO10659 | 21.5 | 16.58 | 3.040E7 | Periplasm |

**Table S3. The 50 most abundant proteins identified by LTQ-Orbitrap MS Spectrometry in the nOMV**

| Protein | Acc.No | MW [kDa] | Coverage | Area | Subcellular location |
| --- | --- | --- | --- | --- | --- |
| outer membrane protein A | ABO13246 | 38.4 | 61.80 | 5.126E9 | Cell outer membrane |
| putative outer membrane protein | ABO11316 | 22.5 | 77.88 | 4.203E9 | Cell outer membrane |
| putative signal peptide | ABO11459 | 41.2 | 73.44 | 2.378E9 | Cell inner membrane |
| bacteriolytic lipoprotein entericidin B | ABO13215 | 4.9 | 50.00 | 2.175E9 | Others |
| putative hemolysin | ABO11749 | 15.7 | 71.53 | 1.633E9 | Cell inner membrane |
| hypothetical protein | ABS90051 | 11.2 | 78.07 | 1.388E9 | Cell outer membrane |
| putative outer membrane protein (OmpH) | ABO12395 | 18.7 | 70.66 | 1.364E9 | Cell outer membrane |
| hypothetical protein | ABO12306 | 17.0 | 60.65 | 8.801E8 | Cell outer membrane |
| peptidyl-prolyl cis-trans isomerase | ABO11972 | 49.0 | 76.38 | 7.926E8 | Cell inner membrane |
| putative signal peptide | ABO12682 | 13.3 | 65.87 | 6.346E8 | Cell outer membrane |
| putative signal peptide | ABO13642 | 12.5 | 10.77 | 6.189E8 | Cell inner membrane |
| putative protein (DcaP-like) | ABO13166 | 47.3 | 14.52 | 6.039E8 | Extracell |
| putative outer membrane protein | ABO13706 | 27.7 | 75.69 | 5.186E8 | Cell outer membrane |
| putative hydrolase | ABO13078 | 35.4 | 68.75 | 4.935E8 | Cytoplasm |
| putative signal peptide | ABO11950 | 21.0 | 75.00 | 4.917E8 | Cell inner membrane |
| putative lipoprotein-34 precursor (NlpB) | ABO13813 | 21.6 | 83.58 | 4.488E8 | Extracell |
| mutarotase precursor | ABO11397 | 41.6 | 60.89 | 3.569E8 | Periplasm |
| peptidoglycan-associated lipoprotein precursor | ABO13012 | 20.1 | 75.40 | 3.568E8 | Cell outer membrane |
| putative outer membrane protein W | ABO10762 | 21.2 | 81.35 | 3.103E8 | Cell outer membrane |
| Cu/Zn superoxide dismutase | ABO13540 | 19.3 | 84.82 | 2.985E8 | Periplasm |
| putative signal peptide | ABO12391 | 32.5 | 56.80 | 2.970E8 | Cell inner membrane |
| hypothetical protein | ABO13757 | 21.0 | 59.89 | 2.914E8 | Periplasm |
| putative lipoprotein | ABO11441 | 12.9 | 28.81 | 2.877E8 | Cell outer membrane |
| putative outer membrane protein | ABO12396 | 95.5 | 48.30 | 2.833E8 | Cell outer membrane |
| putative membrane protein | ABO12960 | 14.7 | 55.07 | 2.794E8 | Cell outer membrane |
| putative signal peptide | ABO11569 | 13.1 | 52.71 | 2.779E8 | Cell outer membrane |
| FKBP-type 22KD peptidyl-prolyl cis-trans isomerase (rotamase) | ABO10542 | 25.1 | 74.89 | 2.672E8 | Cell outer membrane |
| putative lipoprotein precursor | ABO13390 | 18.0 | 52.20 | 2.600E8 | Cell inner membrane |
| hypothetical protein | ABO10610 | 13.6 | 42.97 | 2.449E8 | Cell outer membrane |
| hypothetical protein | ABO11890 | 35.0 | 73.16 | 2.406E8 | Cell inner membrane |
| putative signal peptide | ABO13198 | 18.8 | 58.14 | 2.361E8 | Cell outer membrane |
| glutamate/aspartate transport protein | ABO11918 | 32.0 | 65.32 | 2.264E8 | Periplasm |
| putative Zn-dependent protease with chaperone function | ABO11610 | 27.6 | 47.51 | 2.104E8 | Cell inner membrane |
| putative signal peptide | ABO13522 | 15.6 | 58.74 | 2.051E8 | Extracell |
| putative competence protein (ComL) | ABO11274 | 42.8 | 77.40 | 1.999E8 | Cell inner membrane |
| hypothetical protein | ABO10958 | 41.1 | 51.97 | 1.758E8 | Cell outer membrane |
| disulphide interchange protein (DsbC-like) | ABO13231 | 26.3 | 75.85 | 1.743E8 | Periplasm |
| hypothetical protein | ABS90126 | 14.5 | 39.10 | 1.636E8 | Cell outer membrane |
| hypothetical protein | ABO11009 | 34.5 | 55.27 | 1.597E8 | Extracell |
| lipoprotein precursor | ABO12850 | 29.6 | 63.77 | 1.533E8 | Cell outer membrane |
| putative long-chain fatty acid transport protein | ABO13185 | 53.1 | 16.90 | 1.523E8 | Cell outer membrane |
| putative signal peptide | ABO13547 | 13.7 | 60.66 | 1.475E8 | Cell outer membrane |
| hypothetical protein | ABO12790 | 44.5 | 45.54 | 1.349E8 | Cell inner membrane |
| putative signal peptide | ABO12106 | 10.2 | 38.20 | 1.304E8 | Cell outer membrane |
| putative serine protease | ABO12942 | 49.0 | 45.20 | 1.294E8 | Periplasm |
| putative signal peptide | ABO12909 | 44.0 | 33.33 | 1.286E8 | Cell outer membrane |
| outer membrane lipoprotein | ABO11326 | 14.4 | 79.55 | 1.260E8 | Cell outer membrane |
| FKBP-type peptidyl-prolyl cis-trans isomerase (rotamase) | ABO10543 | 25.9 | 58.33 | 1.223E8 | Cell outer membrane |
| hypothetical protein | ABO10765 | 44.6 | 48.28 | 1.210E8 | Cell outer membrane |
| hypothetical protein | ABO13336 | 12.9 | 70.00 | 1.104E8 | Periplasm |
